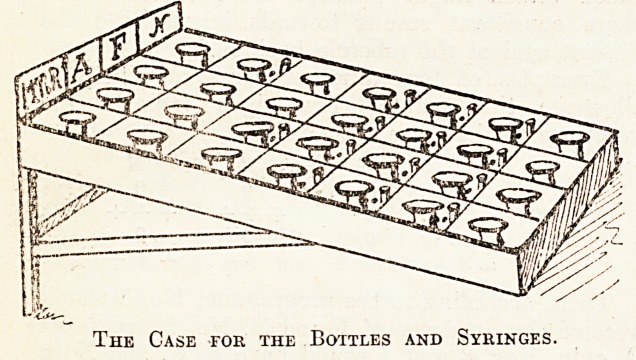# A New Method of Tuberculin Treatment

**Published:** 1915-01-09

**Authors:** Lytton Maitland


### A NEW METHOD OF TUBERCULIN TREATMENT.

### The Deycke-Much Programme in Germany.

By LYTTON MAITLAND, M.D., D.P.H. (Camb.).

Both in the Eppendorf and in the Lübeck general hospitals an extensive trial has been made of certain modifications of tuberculin treatment; the results obtained have been carefully recorded and analysed for a period of two and a half years—that is to say, up to the beginning of the past year. The results of these investigations and a full description of the methods were to have been given at the Tuberculosis Congress which was to have been held in September at Berne. This congress, like many other propositions, has been drowned in the whirlpool of war, so that it may be of interest to give a brief outline of this newest tuberculin treatment. The information was gathered from visits paid to the two hospitals mentioned above, and from the demonstrations of Dr. Ernest Altstaedt, of the Lübeck Hospital. Further details have recently been published in the thirty-first volume of the *Beiträge zur Klinik der Tuberculose*, where the theory and practice of the method are explained in full by Dr. Altstaedt.

The work has necessitated a very large number of serological investigations, for every patient was tested by complement fixation and intracutaneous reactions before deciding the requisite dose in each individual case. The method is generally spoken of as the Deycke-Much treatment. Professor Deycke has charge of a medical division of the Eppendorf Hospital in Hamburg, and is also one of the directors of the Lübeck Hospital, while Dr. Much is the medical superintendent of the institute for experimental therapy at Eppendorf. His name in this country is well known in connection with the granules found in films of the sputum of tuberculous patients. The very lengthy theoretical considerations which lead up to the development of this method cannot here be even summarised; it will suffice to indicate the main details of the method.

### The Emulsion.

By the treatment for some weeks of tubercle bacilli with a 1-per-cent. solution of lactic acid, an emulsion results in which the bacilli as such are unrecognisable and do not stain by Ziehl-Nielsen, nor are Much granules to be observed in the amorphous masses of the deposit. The immunising power of the emulsion is, however, not lost. From this preparation, called M.Tb. (Milchsäure=lactic acid), are separated four constituents as shown in the following diagram:

The three constituents chiefly concerned in this method are A, F, and N. It was found by means of complement fixation tests that individual patients varied considerably in the reactions they gave to each of these substances. All three were capable of inducing the formation of specific antibodies, but some individuals suffering from tuberculosis might react to all three, some to only one or two, and some to none, and, moreover, those that did react would do so in varying degree.

### Immunisation.

The aim of this treatment, which may be described as the Deycke-Much partial antigen method, is to immunise the patient, just as is the aim of any tuberculin treatment. But by treating the patient with that particular antigen against which he possesses deficient antibodies, it is hoped to achieve the object of bringing all his antibodies up to a uniform pitch, as it were, so that a combined attack might produce more constant and more consistent results towards immunising the patient against the tubercle bacillus.

Examples of four different cases may help to illustrate this point. The signs indicate the reactions to the three antigens.

| | A | F | N |
|---|---|---|---|
| No. 1 ... ... | + + + | 0 | 0 |
| No. 2 ... ... | 0 | + + + | + + + |
| No. 3 ... ... | 0 | 0 | 0 |
| No. 4 ... ... | + + + | + + + | + + + |

Then, according to the programme, No. 1 would receive certain doses of F and N, No. 2 would get A only, Nos. 3 and 4 would have A, F, and N in appropriate doses, or even the M.Tb.R. itself.

### Range of Dilutions.

The complement fixation test is a somewhat cumbersome method by which to gauge the reactions of many individual patients, and it was soon found that the intracutaneous reaction could be substituted, and yet the results were strictly comparable. Exceedingly high dilutions were used of each of the antigens, even up to a one hundred thousandth millionth ($\frac{1}{100000000000}$). At one sitting as many as twenty reactions are set out, the outer sides of both arms being used. The usual range of dilutions is as follows:

| | |
|---|---|
| M.Tb.R. ... ... | 1 in 1,000,000 to 1 in 10,000 millions |
| A ... ... | ditto |
| F ... ... | 1 in 1.000 to 1 in 10 millions |
| N ... ... | 1 in 1,000 to 1 in 1 million |

These very dilute solutions require to be kept in bottles specially reserved for each particular dilution, and a separate syringe and needle must be used for each bottle and for that one only, for it was found, no matter how carefully and thoroughly bottles and syringes were cleansed, any interchange of implements always meant vitiated results owing to the extreme dilutions used—the merest trace of another dilution assuming great relative importance. In actual practice twenty bottles are used, arranged in five rows in a wooden case; two other rows of compartments contain reserve bottles (see illustration). The twelve compartments containing the highest dilutions have their own syringes and needles. The results obtained on each patient are recorded graphically by means of squares, so that at a glance can be seen the proportional reactions to each antigen. In practice it is found that the majority of individuals have all three antibodies about equally strong or equally weak; of the minority, the larger number are strongest in F and N and weakest in A, and the remainder are strongest in A. So that the first class are treated with M.Tb.R. or A + F + N; the second class with A, and the third with F + N.

After these reactions have been determined, the patient receives *daily* injections of a percentage mixture of antigens, the *titre* of which is governed by results of the intracutaneous trial injections and by the complement fixation method. The injections are controlled by the closest clinical observations, and are given intramuscularly. The doses are increased by about one-half of the last dose in

The Case for the Bottles and Syringes.

a series such as the following: 0.1, 0.15, 0.2, 0.3, 0.5, 0.75, 1 c.c. The directions next specify a pause of two or three weeks, followed by a repetition of the intracutaneous and complement fixation tests. The new values now obtained form the basis of another series of injections of which the dose of each constituent is regulated by the proportional reactions.

Such is a bare outline of the scheme, and it is obvious that at the outset careful estimations have to be made of the skin reactions after the manner in which the quanti-Pirquet test is employed.

### Results.

Naturally one inquired particularly after the results which had been obtained so far from a method which, to say the least, is a somewhat tedious one. In both hospitals the cases treated fall into the more advanced classes rather than into the early or first stage. Of the third stage of pulmonary disease, there were 141 cases, or 54 per cent.; of the first and second stages, 94 cases, or 36 per cent.; and of tuberculosis of other organs there were 26 cases, or 10 per cent. Of these cases the results were recorded as follows.

| | Number | Percentage | Pulm. Tuber. Stage I–II. | Pulm. Tuber. Stage III. | Tuberculosis Other Organs |
|---|---|---|---|---|---|
| Improved ... | 187 | 72 | 93 % | 54 % | 85 % |
| In statu quo | 34 | 13 | 6 % | 19 % | 11 % |
| Worse ... | 40 | 15 | 1 % | 27 % | 4 % |
| Total ... | 261 | | | | |

The cases were not in any way selected, and even unfavourable cases were included; only the moribund were excluded. In view of this the above figures may be regarded as comparing more favourably than those produced by workers with other methods.

But, it will be admitted, further analyses are necessary before it can be definitely stated that this Deycke-Much programme can achieve results commensurately superior to the trouble and care that the method entails. After all, the human body must be in a position to call up antibodies, and should there be lacking adequate response, then no method of active immunisation can avail to help towards the overthrow of the infection.

Too short a time has elapsed to pronounce a final judgment on the results of this treatment in cases of pulmonary tuberculosis as regards permanent cure, but it is worth noting that in a later set of figures relating to cases who received daily injections no less than 75 per cent. of the Stage III. pulmonary cases are placed in the improved class; while 92 per cent. of cases of tuberculosis of other organs were definitely classed as "greatly improved." The good results are said to be obtained in less than half the time formerly necessary to produce equivalent benefit.

## Figures and Tables

**Figure f1:**